# Impact of peptide:HLA complex stability for the identification of SARS-CoV-2-specific CD8^+^T cells

**DOI:** 10.3389/fimmu.2023.1151659

**Published:** 2023-05-18

**Authors:** Olivia Lie-Andersen, Mie Linder Hübbe, Krishanthi Subramaniam, Daniel Steen-Jensen, Ann Christina Bergmann, Daniel Justesen, Morten Orebo Holmström, Lance Turtle, Sune Justesen, Telma Lança, Morten Hansen

**Affiliations:** ^1^ Immunitrack ApS, Copenhagen, Denmark; ^2^ National Center for Cancer Immune Therapy (CCIT-DK), Department of Oncology, Copenhagen University Hospital, Herlev, Denmark; ^3^ Department of Bioengineering, Technical University of Denmark, Lyngby, Denmark; ^4^ Institute of Infection, Veterinary and Ecological Sciences, University of Liverpool, Liverpool, United Kingdom

**Keywords:** NeoScreen stability assay, affinity, epitope, COVID-19, tetramer, pHLA, PBMC, flow cytometry

## Abstract

Induction of a lasting protective immune response is dependent on presentation of epitopes to patrolling T cells through the HLA complex. While peptide:HLA (pHLA) complex affinity alone is widely exploited for epitope selection, we demonstrate that including the pHLA complex stability as a selection parameter can significantly reduce the high false discovery rate observed with predicted affinity. In this study, pHLA complex stability was measured on three common class I alleles and 1286 overlapping 9-mer peptides derived from the SARS-CoV-2 Spike protein. Peptides were pooled based on measured stability and predicted affinity. Strikingly, stability of the pHLA complex was shown to strongly select for immunogenic epitopes able to activate functional CD8^+^T cells. This result was observed across the three studied alleles and in both vaccinated and convalescent COVID-19 donors. Deconvolution of peptide pools showed that specific CD8^+^T cells recognized one or two dominant epitopes. Moreover, SARS-CoV-2 specific CD8^+^T cells were detected by tetramer-staining across multiple donors. In conclusion, we show that stability analysis of pHLA is a key factor for identifying immunogenic epitopes.

## Introduction

The Severe acute respiratory syndrome coronavirus 2 (SARS-CoV-2) pandemic has highlighted the importance of having tools to rapidly characterize vaccine targets to speed up vaccine development. Moreover, with the emergence of new viral strains (1) and continuous re-infection, despite vaccine-generated antibody-responses, the importance of T cells in anti-viral immunity has become much more clear (2–4). Nevertheless, prediction of immunogenic T cell epitopes based on affinity has a high rate of false positives and it is desirable to narrow down this field of potential epitopes to avoid spending time and resources on non-immunogenic epitopes (5, 6).

Proteins are processed intracellularly by the proteasome for peptides to be presented on the surface by the major histocompatibility complex (MHC), also called human leukocyte antigen (HLA) in humans (7). For a peptide:HLA (pHLA) complex to induce a durable immune response, the peptide needs to bind to a HLA with adequate affinity and stability to permit presentation of the pHLA complex at the cell surface. This pHLA complex has to persist until a circulating T cell containing the cognate T-cell receptor arrives, recognizes the complex, binds to it and initiates activation of the T cell, generating a specific immune response (8). Currently available immunogenicity *in silico* prediction tools, such as NetMHCpan4.0 (9) are based on a large quantity of measured pHLA complex affinity data sets from databases such as Immune Epitope Data Base (IEDB) (10). However, NetMHCpan4.0 and similar tools predict many false positive T-cell epitopes (5, 11). Therefore, there is a need to create tools able to narrow down the predicted peptides thus enriching for truly immunogenic epitopes. Strong experimental evidence supports the notion that stability of the pHLA complex is a better indicator of immunogenic epitopes than predicted affinity (6, 12–14).

Here we investigated whether stability of the pHLA complex could improve the discovery of immunogenic epitopes, by screening overlapping 9-mer peptides from the spike SARS-CoV-2 protein on three common HLA class I-alleles: HLA-A*0101, HLA-A*0201 and HLA-A*0301. Peptides were divided into two groups of either 1) high measured pHLA complex stability with varied predicted affinity, named hereafter High Stab or 2) high predicted affinity epitopes with low measured pHLA complex stability, named hereafter Low Stab. Next, the presence of peptide-specific T cells in HLA-matching SARS-CoV-2 vaccinated and convalescent donors was compared. We observed a significant enrichment of validated epitopes in the High Stab peptide pool in comparison with the Low Stab pool and therefore we conclude that high stability of the pHLA complex across several HLA alleles is a key factor for epitope discovery.

## Methods

### Samples and isolation of peripheral blood mononuclear cells (PBMCs)

The vaccinated cohort of donors were vaccinated through the Danish government supplied vaccination program targeting the SARS-CoV-2 spike protein. The cohort had received a mix of vaccines, both mRNA and adenovirus based. Samples were collected 25-150 days after end of vaccination. Donors were in the ages from 27-50 and both male and female (**Figure S1A**). The project was approved by the Regional Ethics Committee, Capital Region, Denmark (approval number: H-22046811). Blood was collected from vaccinated volunteers after informed consent. Whole blood was collected in Lithium Heparin coated collection tubes and PBMCs isolated by density gradient centrifugation. The PBMCs were washed twice in PBS for 5 min at 300g, where after they were counted and frozen in 90% Fetal Bovine Serum (FBS) and 10% dimethyl sulfoxide (DMSO) and kept at -150°C. Isolated PBMCs from SARS-CoV-2 convalescent donors were obtained from the University of Liverpool Acute Virus Immunity Study from consenting donors (AVIS, ethics approval ref 16/NW/0170) and kept at -150°C upon arrival. Samples from convalescent donors were collected in the period 5^th^ of May 2020-26^th^ of March 2021 and 1-10 months after positive PCR test (**Figure S1B**).

### HLA typing

HLA typing was conducted at IMGM Laboratories GmbH, Germany. Genomic DNA isolation and high-resolution HLA typing of HLA–A, -B, -C, -DRB1, -DQB1 and -DPB1 was carried out in collaboration with their strategic partner and sister company MVZ Martinsried GmbH in Germany (**Table S2**, **S3**).

### Affinity prediction of spike peptides

Affinity prediction of the spike protein was carried out. The amino acid sequences were retrieved from “Wuhan seafood market pneumonia virus isolate Wuhan-Hu-1, complete genome. ACCESSION: MN908947 VERSIONMN908947.3 NetMHCpan4.0 (9) was used to predict the peptides most likely to bind to HLA-A*0101, -A*0201 and -A*0301. The cut off value for a binder was K_D_ 500 nM.

### Peptides and NeoScreen^®^ stability assay

As previously described in Prachar et al. (11) 1286 overlapping spike peptides for NeoScreen^®^ were synthesized by Intavis by the SPOT method using Fmoc-based chemistry on cellulose membranes and cleaved from solid support. 5% of the crude peptides were analyzed by MALDI-TOF to confirm the presence of a peptide with the correct molecular weight. Peptides were produced as crudes with an anticipated purity of 50%-70%. Peptides for peptide pools were produced in house by solid phase synthesis using Fluorenylmethyloxycarbonyl protecting group (Fmoc)/Tert-butoxycarbonyl protecting group (Boc) chemistry and were produced as C terminal amides. Afterwards, the peptides were dissolved in DMSO (10 mM) and stored at -20°C.

NeoScreen^®^ stability assays of the 1286 overlapping peptides were carried out by mixing the peptides with HLA I molecules to a final concentration of 2-10 nM and with a peptide concentration of 1 µM. Known T-cell epitopes were used as a reference (HLA-A*0101: VTEHDTLLY, -A*0201: LLFGYPVYV, -A*0301: AVAHKVHLMYK). After 24 hours of incubation, the solutions were distributed into 384 well plates and urea added at 0, 2, 4 and 6M. Plates were developed in a conventional ELISA described previously (15, 16) using a conformational specific mouse anti-human w6/32 antibody (Invivoma) and goat anti-mouse Fc HRP (Sigma-Aldrich). Stability of individual peptides was calculated relative to the reference peptides; therefore, stability is calculated as a percentage. The threshold for considering a high stability binder was 50% or higher.

### Long-term peptide stimulation of PBMCs

PBMCs were thawed in warm X-VIVO 15 medium (Lonza) and washed twice before seeding at 1.5-3 x 10^6^ cells/well into 48-well plates in 1 ml. Peptide pools were added at 5-10 uM and incubated on the bench wrapped in parafilm for 2 hours before 1 ml X-VIVO + 10% human serum (HS) (Sigma-Aldrich) was added and the PBMCs were incubated at 37°C with 5% CO_2_ in a humidified atmosphere. On day 1, 120 U/ml IL-2 (Proleukin) was added. The cells were incubated for 12-14 days with media change on day 10 if necessary. The cells were counted before harvest.

### Intracellular cytokine staining

PBMCs were washed (300g, 5 min) in warm RPMI-1640 (Life Technologies) by centrifugation. Cells were seeded in a round bottom 96-well plate in duplicate at ~0.5 x 10^6^ cells/well and stimulated with 5uM peptide pools and 0.1μg/ml anti-CD28 (BD Biosciences) and anti-CD49d (BD Biosciences) for 1h, before adding GolgiPlug, GolgiStop, and anti-CD107a-BV421 (all BD Biosciences) and incubated for another 5 hours. Afterwards, the cells were transferred to a 96-v-shaped plate and washed (5min, 300g) twice in FACS buffer (FB) (PBS, 2mM EDTA, 0,05%NaN_3_, 0.5% bovine serum albumin (Sigma-Aldrich)). The cells were stained for 10 min at 4°C with Invitrogen™ LIVE/DEAD™ Fixable Near-IR Dead Cell Stain Kit, before pre-titrated surface antibodies [BV510-CD4, APCR700-CD8, BV786-CD3, BV605-CD56 (BD Biosciences)] were added and incubated for 20 additional min at 4°C. The cells were washed (5min, 300g) with cold PBS and permeabilized and fixed with Fixation/Permabilization kit (ebiosciences) in the dark for 30 min at room temperature (RT) according to the protocol provided by the supplier. The cells were washed (5min, 300g) in freshly prepared permeabilization buffer (ebioscience) and stained with intracellular antibodies [APC-TNFa, PE-Cy7-IFNy, PE-CD137 (BD Biosciences)] for 20 min at 4°C. The cells were washed (5min, 300g) twice in permeabilization buffer, before acquisition on a NovoCyte Quanteon Flow Cytometer (Agilent Technologies).

### Tetramers

Tetramers were produced in-house by Immunitrack, as described previously in Leisner et al, (17). Briefly, for HLA-I tetramers *E.coli* produced heavy chains were diluted into a reaction buffer (50mM tris-maleate pH 6.6, 0,1% pluronic F86 NF (BASF, a surfactant compatible with cellular use), with excess β2-microglobulin and excess peptide of interest and incubated at 18 degrees for 48h. For tetramerization, 1:4 molar ratio of Streptavidin-APC or Streptavidin-PE to the peptide-HLA monomers was added sequentially over 1h. The resulting PE- or APC-tetramers were stored at -20°C before use.

Tetramer staining was conducted by washing long-term stimulated PBMCs in FB (300g, 5 min). Cells were seeded in a v-shaped 96-well plate. Afterwards, were the cells stained with 1:50 dilution tetramer conjugated with APC and PE, 1:1000 Invitrogen™ LIVE/DEAD™ Fixable Near-IR Dead Cell Stain Kit and 100 nM dasatinib and incubated for 15 min at 37°C. Then surface antibodies were added [BV786-CD3, APCR700-CD8, BV510-CD4, BV605-CD56 (BD Bioscience)] and incubated for additional 20 min. Afterwards the cells were washed (300g, 5min) twice in FB before acquired on flow cytometer (Novocyte Quanteon, Agilent Technologies).

### Statistical analysis

All graphs were generated in GraphPad prism v9.3.1 and RStudio version 1.2. All bar graphs are depicted with mean ± standard error of the mean.

## Results

### Selection of SARS-CoV-2 spike peptides based on stability measurements and affinity predictions

**Figure 1:**
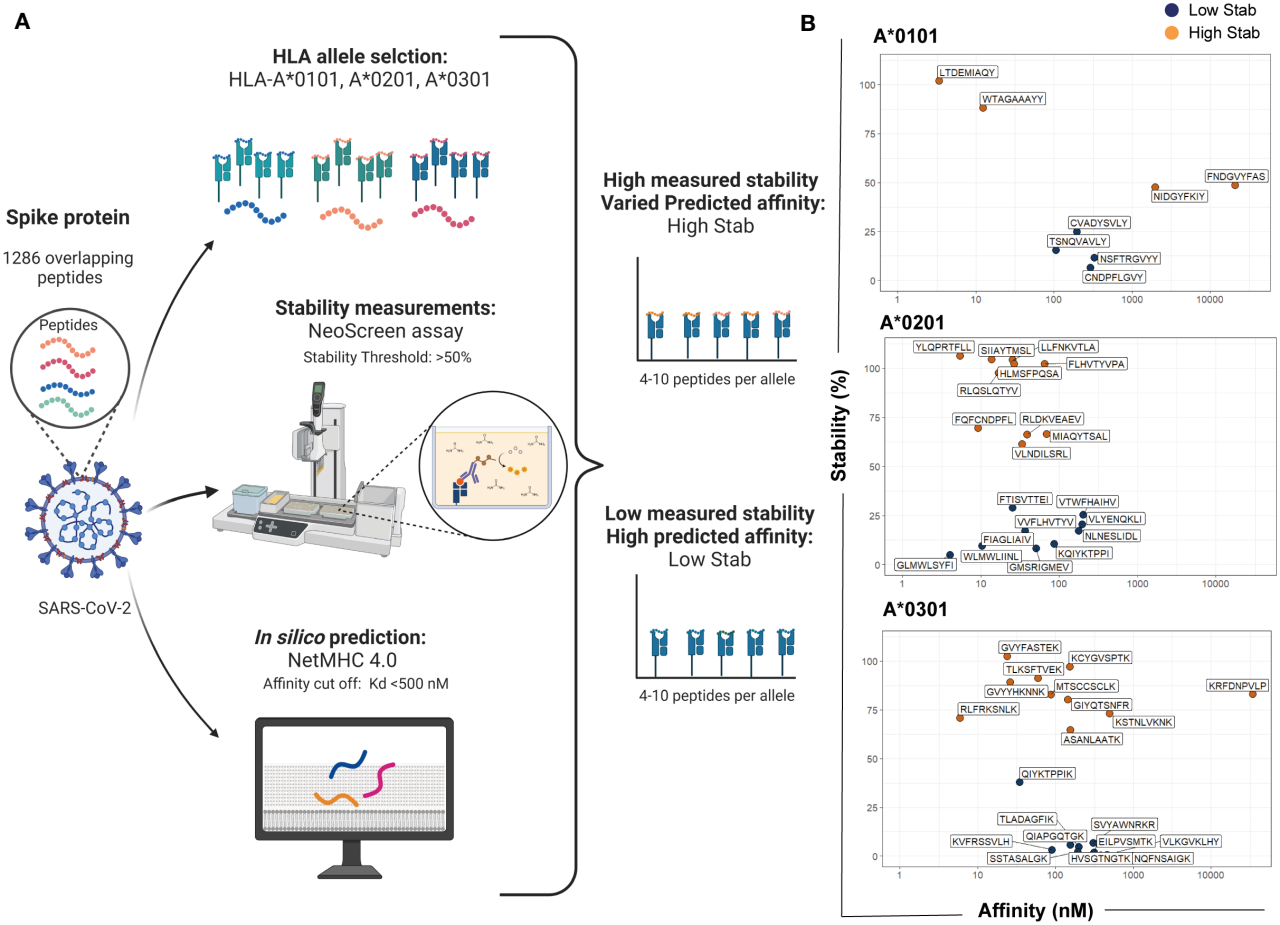
Selection of SARS-CoV-2 spike peptides based on stability measurements and affinity predictions. Workflow for selecting and grouping peptides for the experiment design. **(A)** An overlapping 9-mer peptide library from the spike protein (total of 1286 peptides) was analyzed for stability using NeoScreen^®^ assay and the same peptides were analyzed with NetMHC4.0 for affinity predictions. Assays and predictions were done in the context of HLA-A*0101, -A*0201 and -A*0301. The figure was created in BioRender. **(B)** For each allele, peptides were divided in two groups named High Stab for peptides with high measured stability (>50%) and varied predicted affinity (orange dots) or Low Stab for peptides with low measured stability (<50%) and a predicted affinity for binders (<500nM) (blue dots).

To address whether pHLA stability is a better discriminator of immunogenicity than affinity, the spike protein from SARS-CoV-2 was chosen as a model due to its importance as a vaccine target. **Figure 1A** depicts an overview of the workflow used for selecting and grouping peptides for the experiment design. HLA-I molecules preferentially bind to 9-mer peptides (14, 18). Therefore, an overlapping 9-mer peptide library of the spike protein sequence was analyzed. Our in-house stability assay, termed NeoScreen^®^, was employed to investigate the stability of the pHLA complex formed between the 1286 peptides from the 9-mer overlapping-peptide library to the three common HLA-I alleles in Europe, HLA-A*0101, -A*0201 and -A*0301. Briefly, in the NeoScreen^®^ assay, peptides were incubated with HLA-I to obtain complexes. After incubation, the folded complexes were subjected to stress by exposure to increasing urea concentrations followed by analysis with w6/32, a pan HLAclass I conformational-specific antibody. A reference peptide for each allele was included to ensure assay performance, and stability measurements made with this reference peptide were used as a relative value (100%) for stability assessments of individual peptides.

We considered a stable binder to be a peptide with a threshold of at least 50% compared with the reference peptide on the pHLA of the correspondent allele (**Figure S2A**). Affinity of the pHLA was predicted using *in silico* tool NetMHCpan 4.0 (9). Peptides were selected based on the top predicted binders with a cut-off value for the equilibrium dissociation constant, K_D_, below 500 nM (**Figure S2B**). Then, peptides were divided into two groups named High Stab for peptides with high measured stability (>50%) and varied predicted affinity or Low Stab for peptides with low measured stability (<50%) and a predicted affinity for binders (<500nM) (**Figure 1B** and **Table S1**).

### Stimulation with High Stab peptide pools enriches for spike-specific-CD8^+^T cells in a vaccinated cohort

**Figure 2:**
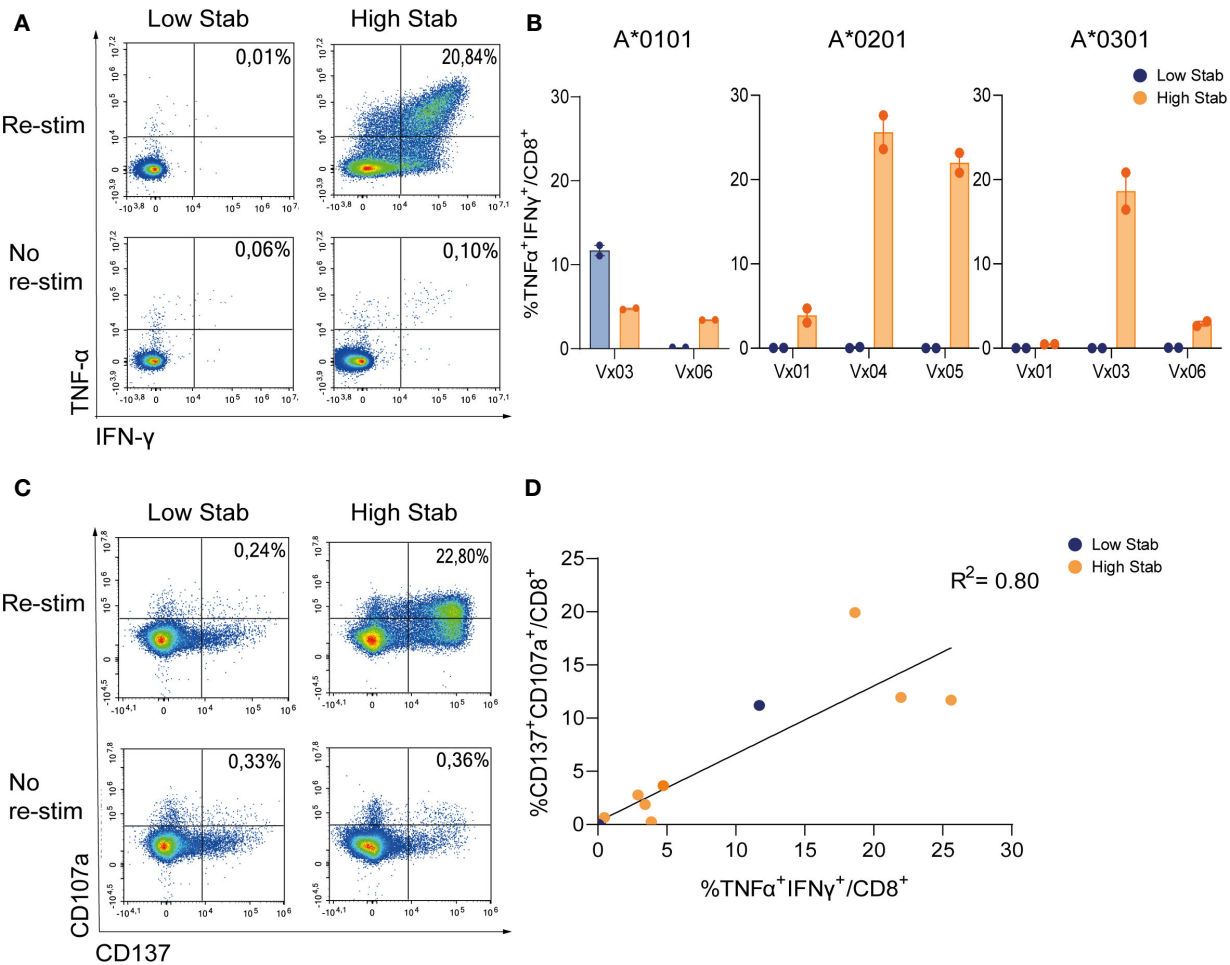
Stimulation with High Stab peptide pools enriches for spike-specific-CD8^+^T cells in a vaccinated cohort. PBMCs from vaccinated (Vx) donors were stimulated for 12-14 days in the presence of low dose interleukin (IL)-2, with High Stab or Low Stab peptide pools followed by an overnight re-stimulation with the same peptide pools. Surface and intracellular cytokine stain was performed, and cells were analyzed by flow cytometry **(A)** Representative plot of interferon (IFN)-γ and tumor necrosis factor (TNF)-α on CD8^+^T cells after Low Stab peptide stimulation (left) or High Stab peptide stimulation (right), with (top) or without (below) re-stimulation. **(B)** Percentage of IFN-γ and TNF-α double positive CD8^+^ T cells on HLA-A*0101, -A*0201, -A*0301 donors. Samples stimulated with Low Stab peptide pools are depicted in blue and samples stimulated with High Stab peptide pools are depicted in orange. Graphs shows mean ± standard error of the mean of technical duplicates representing three independent experiments. **(C)** Representative plot of activation marker CD137 and degranulation marker CD107a on CD8^+^T cells after Low Stab peptide stimulation (left) or High Stab peptide stimulation (right), with (top) or without (below) re-stimulation. **(D)** Correlation between CD137^+^CD107a^+^ double positive and TNF-α^+^IFN-γ^+^ double positive CD8^+^ T cells in all donors analyzed regardless of the HLA typing.

To compare CD8^+^T cell specificity towards the two groups of peptides, PBMCs from six donors vaccinated with COVID-19 vaccines were processed and HLA-typed (**Table S2**). PBMCs were stimulated with peptide pools and expanded for 12-14 days in the presence of low dose interleukin (IL)-2, before re-stimulation with peptide pools and examined for release of tumor necrosis factor (TNF)-α and interferon (IFN)-γ, as well as induction of activation marker CD137 and degranulation marker CD107a. In HLA-A*0201^+^ and -A*0301^+^ donors, after stimulation with High Stab peptide pools we detected a clear IFN-γ and TNF-α response in CD8^+^T cells (**Figures 2A, B**), which were not observed with Low Stab peptide pools, with one exception for HLA-A*0101. For all alleles, cytokine responses were correlated with surface expression of CD137 and CD107a (**Figures 2C, D**).

These data showed that the measured stability of the pHLA complexes correlated highly with immunogenicity. We observed an enrichment of responses in High Stab peptide pools cultures as compared to Low Stab pools for all alleles, with 2/2 responders for HLA-A*0101, 3/3 responders for HLA-A*0201 and 2/3 responders for HLA-A*0301. Interestingly however, for one HLA-A*0101^+^ donor, (Vx03) expression of IFN-γ and TNF-α was seen in response to both High Stab and Low Stab peptide pools.

### Stimulation with High Stab peptide pools enriches for spike-specific-CD8^+^ T cells in a convalescent cohort

**Figure 3:**
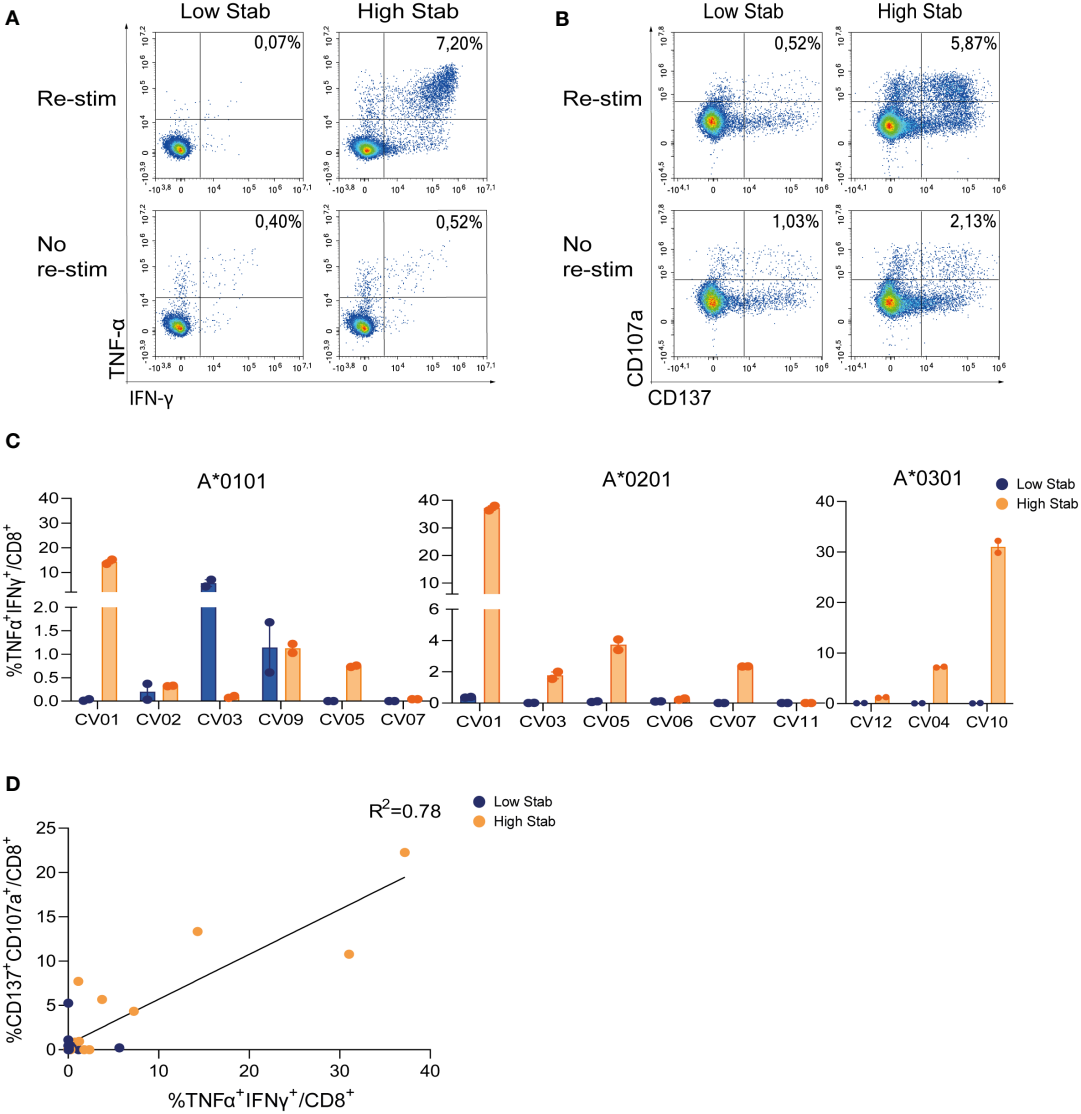
Stimulation with High Stab peptide pools enriches for spike-specific-CD8^+^ T cells in a convalescence cohort. PBMCs from convalescence (CV) donors were stimulated for 12-14 days in the presence of low dose interleukin (IL)-2, with High Stab or Low Stab peptide pools followed by an overnight re-stimulation with the same peptide pools. Surface and intracellular cytokine stain was performed, and cells were analyzed by flow cytometry **(A)** Representative plot of interferon (IFN)-γ and tumor necrosis factor (TNF)-α on CD8^+^T cells after Low Stab peptide stimulation (left) or High Stab peptide stimulation (right), with (top) or without (below) re-stimulation. **(B)** Representative plot of activation marker CD137 and degranulation marker CD107a on CD8^+^T cells after Low Stab” peptide stimulation (left) or High Stab peptide stimulation (right), with (top) or without (below) re-stimulation. **(C)** Percentage of IFN-γ and TNF-α double positive CD8^+^ T cells on HLA-A*0101, -A*0201, -A*0301 donors. Samples stimulated with Low Stab peptide pools are depicted in blue and samples stimulated with High Stab peptide pools are depicted in orange. Graphs shows mean ± standard error of the mean of technical duplicates representing two independent experiments. **(D)** Correlation between CD137^+^CD107a^+^ double positive and TNF-α^+^IFN-γ^+^ double positive CD8^+^ T cells in all donors analyzed regardless of the HLA typing.

To validate our previous findings on the vaccination cohort, a similar analysis was performed in eleven HLA-typed SARS-CoV-2 convalescent donor PBMCs. IFN-γ and TNF-α responses (0.7-38%) in CD8^+^ T cells were observed in 3/6 HLA-A*0101, 4/6 HLA-A*0201 and 2/3 HLA-A*0301 donors in response to stimulation with High Stab peptide pools. Responses were observed for Low Stab peptide pools in only two donors with HLA-A*0101 (**Figures 3A, C**). In addition, cytokine responses correlated with upregulation of CD137 and CD107a (**Figures 3B, D**) on CD8^+^ T cells in response to High Stab peptides but not to Low Stab peptides. This might suggest that specific CD8^+^ T cells recognizing Low Stab peptides can be found, but these specific T cells are not fully activated and as responsive as CD8^+^ T cells recognizing High Stab peptides.

Overall, these results across three common HLA class I alleles support the importance of stability of the pHLA complex epitope discover studies for vaccine design or other forms of immunotherapy.

### Deconvolution of peptide pools reveals that spike specific-CD8^+^ T cells recognize a limited number of dominant epitopes

**Figure 4:**
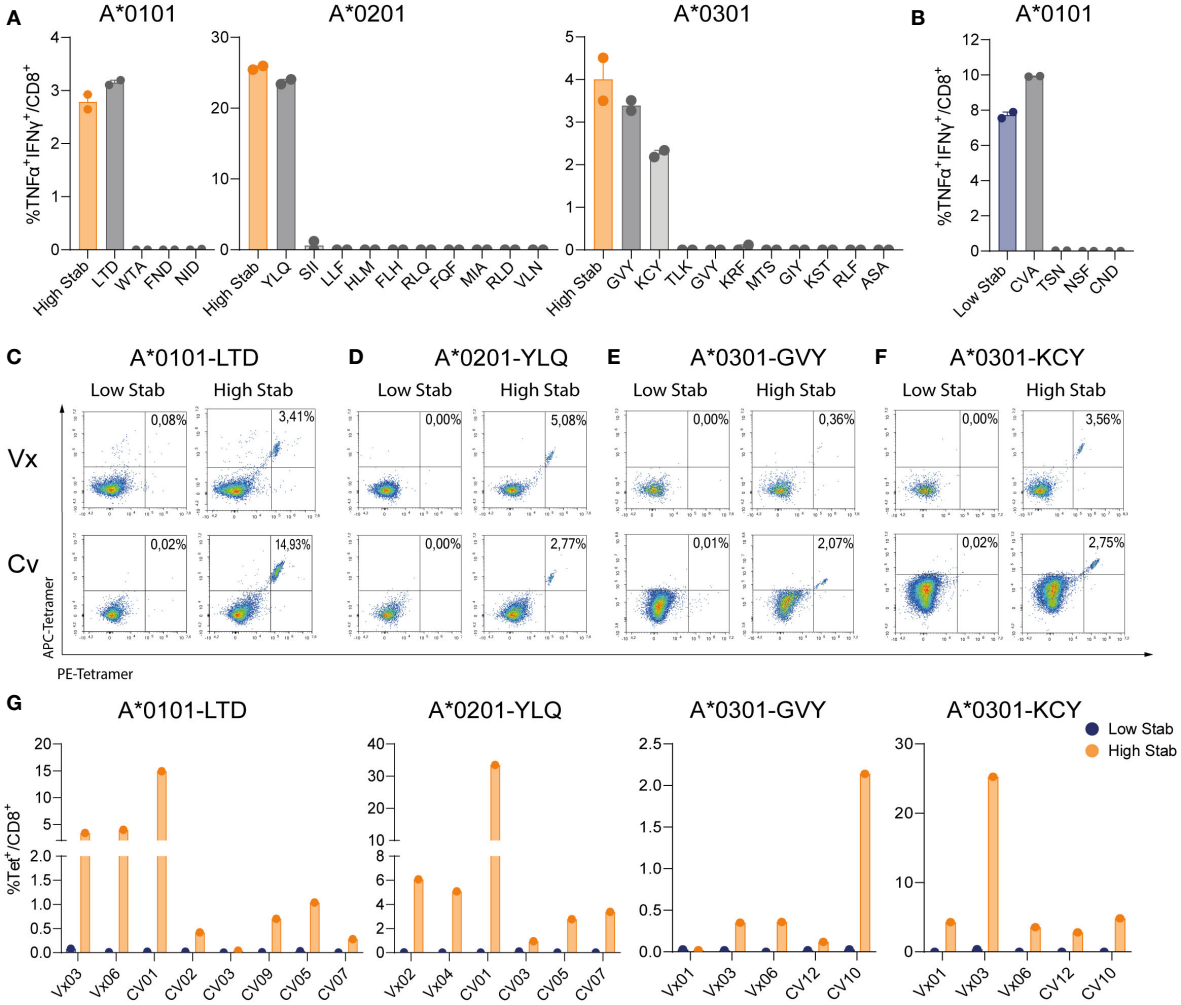
Deconvolution of peptide pools reveals that spike specific-CD8^+^ T cells recognize 1 or 2 dominant epitopes. PBMCs from vaccinated (Vx) and convalescent (CV) donors were stimulated for 12-14 days, in the presence of low dose interleukin (IL)-2, with peptide pools followed by an overnight re-stimulation with individual peptides corresponding to each allele. **(A)** Percentage of TNF-α^+^IFN-γ^+^ double positive CD8^+^ T cells in donor Vx03 HLA-A*0101, donor Vx04 HLA-A*0201 and donor CV10 HLA-A*0301 after re-stimulation with High Stab peptide pool as a control (orange bar) or individual peptides (grey bars). **(B)** Percentage of TNF-α^+^IFN-γ^+^ double positive CD8^+^ T cells in donor Vx03 HLA-A*0101, after re-stimulation with Low Stab peptide pool as a control (blue bar) or individual peptides (grey bars). **(A, B)** Graphs shows mean ± standard error of the mean of technical duplicates representing one experiments. **(C–F)** Representative tetramer stains plots on vaccinated donors (top), convalescent donors (bottom), re-stimulated with Low Stab peptide pools (left) and High Stab peptide pools (right). **(C)** donor Vx03 and CV01 which are HLA-A*0101^+^ stained LTDMIAQY (LTD)-tetramer **(D)** donor Vx04 and CV05 which are HLA-A*0201^+^ stained with YLQPRTFLLV (YLQ)-tetramer **(E)** donor Vx06 and CV10 which are HLA-A*0301^+^ stained with GVYFAKSTEK (GVY)-tetramer **(F)** donor Vx06 and CV12 which are HLA-A*0301^+^ stained with KCYGVSPTK (KCY)-tetramer **(G)** Quantification of tetramer stain (double positive APC and PE) CD8^+^ T cells as in **(C–F)** for multiple Vx and CV donors. Graphs represent two or three independent experiments.

To identify single peptides responsible for the CD8^+^ specific T cell responses, we deconvoluted responses to peptide pools by re-stimulating peptide pool-expanded CD8^+^ T-cells with individual peptides. Deconvolution assays were performed on one donor per allele towards High Stab peptide pools (as a control) and towards four (HLA-A*0101), ten (HLA-A*0201) and ten (HLA-A*0301) single peptides. For HLA-A*0101, we found a TNF-α^+^IFN-γ^+^ CD8^+^ T cell response towards LTDEMIAQY (LTD) and for HLA-A*0201 we detected CD8^+^ specific T cells recognizing YLQPRTFLLV (YLQ). Interestingly, for both alleles, the magnitude of the response towards the peptide pool was equivalent to the magnitude of the response against the single peptides, indicating that these peptides represent the dominant specificities driving the response in these donors (**Figure 4A**). Deconvolution of the immune response recognizing High Stab peptides for the HLA-A*0301, revealed two peptides, KCYGVSPTK (KCY) and GVYFAKSTEK (GVY) as dominant for this allele in this donor (**Figure 4A**).

Since we observed a strong CD8^+^ specific T cell response towards Low Stab HLA-A*0101 peptide pool in donor Vx03 (**Figure 2B**), we also deconvoluted this peptide pool. We observed that the dominant peptide was CVAYSVLY (CVA) that induced a response of the same magnitude as the entire Low Stab pool (**Figure 4B**). Moreover, this response correlated with CD137 and CD107a upregulation on the CVA specific CD8^+^ T cells (data not shown). These results contradicted our other observations; however, CVA was described in Saini at al (19). as a binder for HLA-B*1501 and revealed to be an immunodominant epitope in COVID-19 patients. Notably, donor Vx03 expresses the HLA-B*15:01 allele (**Table S3**), and therefore this is the likely explanation why we see a specific response towards the CVA peptide, which is not stable in a complex with HLA-A*0101 but could be stable in a complex with HLA-B*1501, which we did not test.

To further confirm these data, tetramers were produced for the dominant High Stab epitopes for the three alleles and a larger number of vaccinated and convalescent donors were screened. HLA-A*0101 restricted LTD-tet^+^ CD8^+^ T cell populations were identified in 7/8 donors (**Figures 4C, G**) in proportions equivalent to the immune response previously observed by ICS (**Figure 4A**). This was also the case for HLA-A*0201 donors, where YLQ-CD8^+^ T cell-specific populations were detected in all donors tested (6/6) (**Figures 4D, G**). In HLA-A*0301 donors, both GVY- (**Figures 4E, G**) and KCY-tetramers (**Figures 4F, G**) permitted identification of epitope specific CD8^+^ T cells in 4/5 and 5/5 donors respectively. However, a larger magnitude of KCY-tet^+^ CD8^+^ T cell population (ranging from 2.8-25.3%) than GVY-tet^+^ cells (ranging from 0.12-2.14%) were observed.

In summary, 1 or 2 stable epitopes explained the specific CD8^+^ T cell reactivity per allele. Strikingly, specific responses were seen in 80-100% of the tested donors, which demonstrates that stability is an important factor to consider in the process of epitope discovery.

## Discussion

A vaccine design strategy directed towards inducing both an antibody production and a broad T cell-response could provide the coverage needed to sufficiently protect against emerging pandemic viral strains (2). This requires the accurate identification of true immunogenic T-cell epitopes. Often, epitope discovery is carried out by using *in silico* affinity-based prediction tools. However, this approach gives a high false discovery rate (2, 6). Examining the stability of the pHLA complex provides an alternative to currently available predicted affinity-based models and improves the accuracy of identification of true immunogenic epitopes (13, 14). Traditional stability assays can be quite laborious (20), involving circular dichroism (21), thermal denaturation of the pHLA-I and measurements of the dissociations rates of pHLA using anti-β2-microglobulin (β2m) probes, which is not amenable for high-throughput assays (12, 13). To circumvent this, high-throughput stability assays have been developed, such as the scintillation proximity assay, which utilizes labeled β2m to determine the dissociation rate of the pHLA complex (22, 23). However, this assay involves radioactive β2m, giving rise to safety concerns. We have developed a high-throughput NeoScreen^®^ stability assay which avoids many of the difficulties encountered with traditional stability assays. With NeoScreen^®^ we can determine the decay of the pHLA under denaturing conditions using increasing concentrations of urea, which is safe, accessible, and suitable for high-throughput screening. Furthermore, a known stable immunogenic peptide for each allele is included as a reference, which allows for high reproducibility and comparison between alleles and assays.

In this work, we used NeoScreen^®^ to identify immunogenic epitopes of the SARS-CoV-2 spike protein for three HLA-alleles most common in Europe, HLA-A*0101, -A*0201 and -A*0301. PBMC stimulation with High Stab peptide pools resulted in consistent activation of CD8^+^ T cells. This was observed for all alleles tested in a small group of COVID-19 vaccinated donors and in a larger group of convalescent individuals, indicating that pHLA stability accurately predicts both vaccine and infection induced T cell responses. These results corroborate a previous study where a direct comparison between high and low stability peptides was done in donor PBMCs, with peptides derived from several viral proteins (24), showing a correlation between stability of pHLA and immunogenicity of viral epitopes.

In most studies a peptide is considered immunodominant if found in more than 50% of donors (19, 25) with different magnitudes of response. Although in our data we are enriching for magnitudes by peptide driven expansion, *de novo* responses are not likely to occur solely by this method and we are thus detecting pre-existing CD8^+^ T cell responses towards spike peptides (26). One way to demonstrate that responding CD8^+^ T cells are in fact antigen-experienced T cells would be to enrich for T cells with a central memory or effector memory phenotype prior to *in vitro* stimulation. Due to sparse amounts of donor material for this study, we did not perform this initial selection. We are however confident that we are detecting pre-existing CD8^+^ T cell responses towards spike peptides using this protocol, which has previously been demonstrated for other antigens (27). It is notable that, for the alleles tested in the High Stab peptide pool group, this expansion step resulted in detection of responses in 66-100% of the donors – higher than previous studies. Moreover, we confirmed these results with single epitope stimulation and with tetramer staining and found responses in 80-100% of donors.

For the HLA-A*0101 allele, the dominant epitope was LTDEMIAQY, which was the peptide with highest measured stability, but interestingly also the one with highest predicted affinity. Accordingly, this response was also found in studies where CD8 responses were investigated for this allele based on affinity predictions (19, 28).

It is not uncommon that the predicted affinity of a pHLA complex is different than the measured affinity of the same pHLA complex. The focus of this study was to compare a universally available and commonly used method for epitope selection (*in silico* predictions) to the stability-measurement platform (NeoScreen^®^) developed by Immunitrack. It is possible that including the measured affinity of pHLA complexes would enrich for immunologic epitopes as well, when compared to only predicted pHLA affinity. However, this comparison would add to the cost and burden of experimental work needed to identify epitopes and was therefore not within the scope of this study, hence pHLA affinity measurements are not included in this manuscript. From the data presented, we conclude that pHLA stability strongly selects for immunogenic epitopes and it is likely this selection could be even stronger if additional parameters are added, for example measured affinity.

In the present study, we show that measured stability is a better predictor of immunogenicity than *in silico* predicted affinity for several HLA alleles. Screening for specific spike-CD8^+^ T cell responses based on stability, yielded a significantly lower false discovery rate in both vaccinated and convalescent donors. In summary we demonstrate the significance of investigating stability of the pHLA for determining T cell epitope immunogenicity.

Vaccines that are specifically designed to elicit T cell responses as well as antibody responses may have certain advantages over those that generate antibody responses alone. SARS-CoV-2, for example, has repeatedly escaped the human antibody response, but T cell responses are much less affected by viral variation (29). Although T cell responses probably cannot generate widespread sterilizing immunity, they are nevertheless protective, and reduce disease severity (3, 30). Using techniques such as pHLA complex stability to identify immunogenic T cell epitopes therefore has the potential to improve vaccine design.

## Data availability statement

The original contributions presented in the study are included in the article/**Supplementary Material**. Further inquiries can be directed to the corresponding author.

## Ethics statement

The studies involving human participants were reviewed and approved by Regional Ethics Committee, Capital Region, Denmark (approval number: H-22046811) and Acute Virus Immunity Study (AVIS), United Kingdom, ethics approval ref 16/NW/0170. The patients/participants provided their written informed consent to participate in this study.

## Author contributions

The study was designed by OL-A, SJ, MLH, TL, MH. The experiments were performed by OL-A, SJ, DJ, DS-J, AB. MOH, KS and LT provided critical sample material. All authors contributed to the article and approved the submitted version.
