# Supplementary material for: Impact of peptide:HLA complex stability for the identification of SARS-CoV-2-specific CD8+T cells
Source: Front Immunol. 2023 May 18;14:1151659. doi: 10.3389/fimmu.2023.1151659 (PMC10232890; doi:10.3389/fimmu.2023.1151659)
Supplement: Supplementary file 1 [file DataSheet_1.pdf]

Supplementary Material

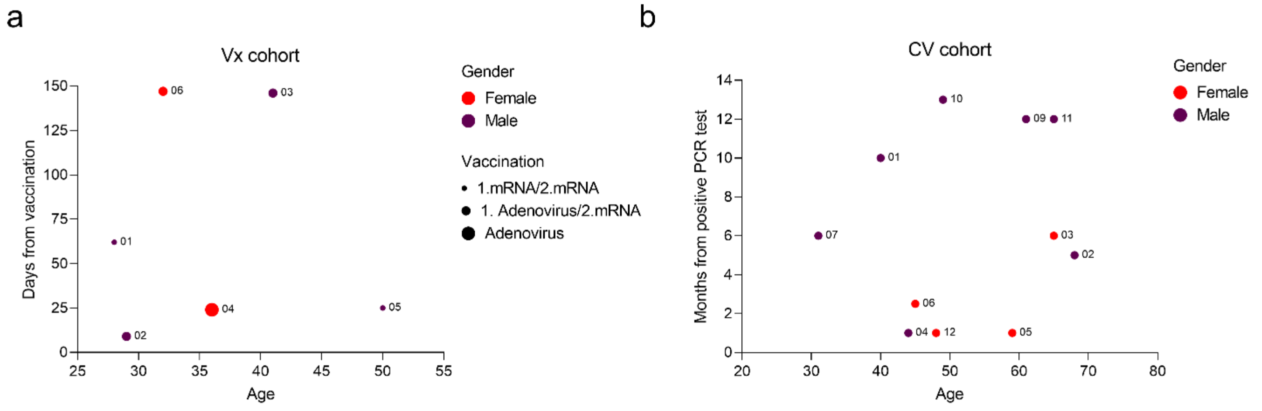

Supplementary Figure 1: Overview of **a)** vaccinated (Vx) and **b)** convalescent cohort (Cv).

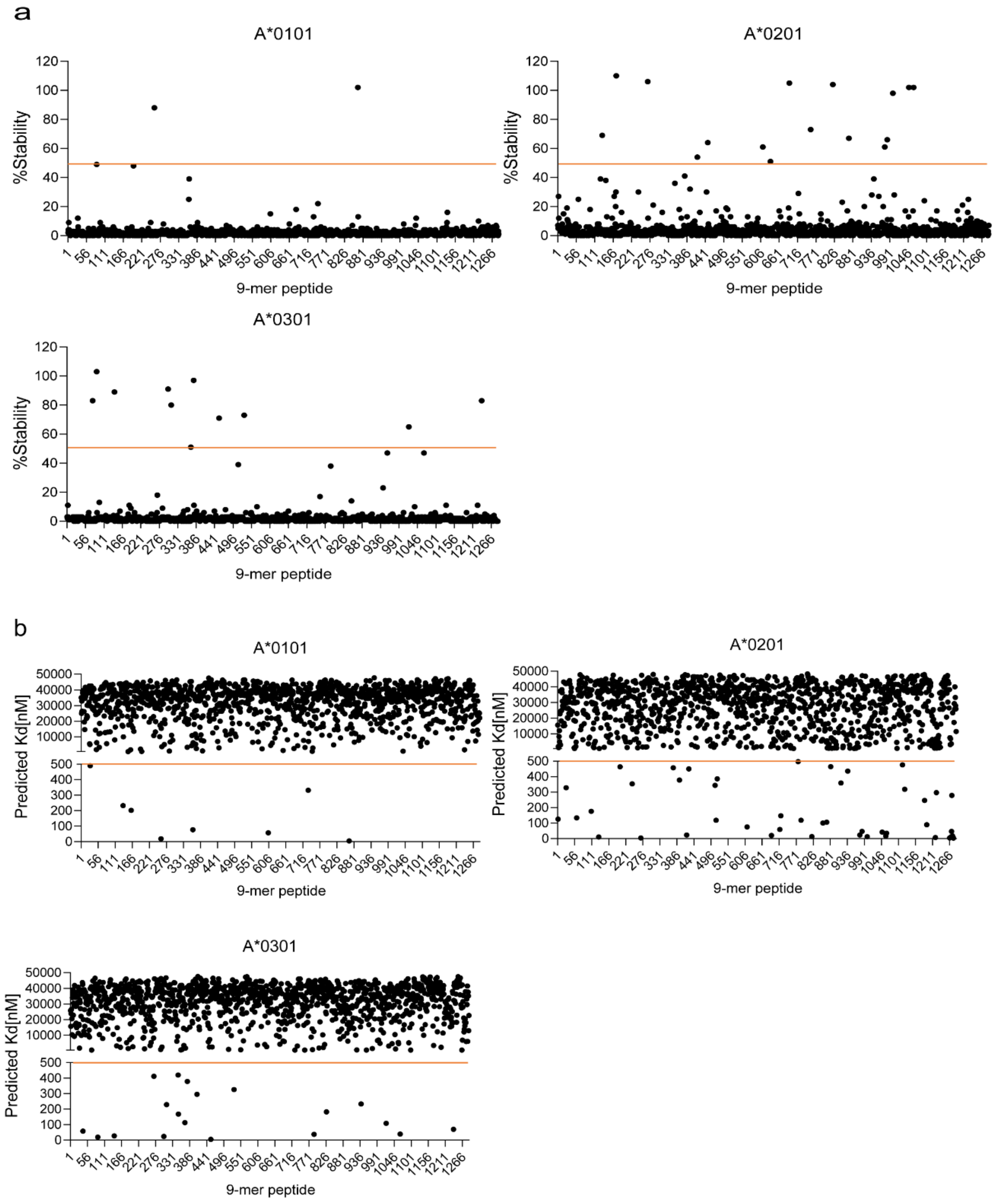

Supplementary Figure 2: Measured stability and predicted affinity of 9-mer library from spike protein (total 1286 peptides). **a)** Measured stability using NeoScreen® in HLA-A*0101, HLA-A*0201 and HLA-A*0301. The red line depicts the 50% cutoff which is relative to a reference peptide (stable binder) for each allele. **b)** *In silico* affinity prediction using NetMHC4.0 for HLA-A*0101, HLA-A*0201 HLA-A*0301.

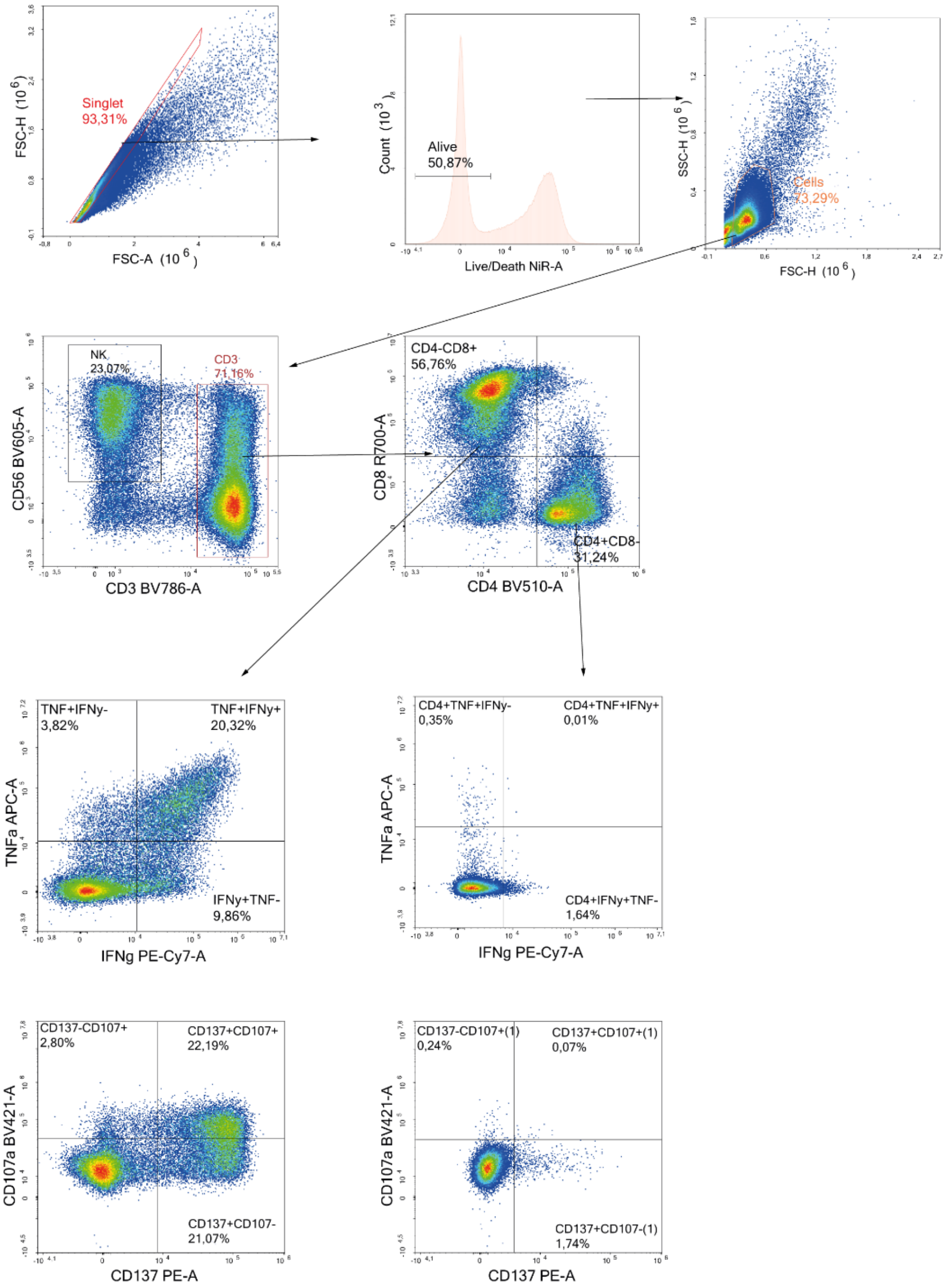

Supplementary Figure 3: Representative gating strategy to identify $CD8^+$ TNF-α^+ IFN-γ^+ T cells and $CD8^+$ $CD107a^+$ $CD137^+$ T cells after long-term peptide stimulated PBMCs.

Supplementary Table 1: List of peptides which are part of the High Stab pool and Low Stab pool for each of the three studied alleles HLA-A*0101, -A*0201 and -A*0301. For each peptide, the measured stability (relative to reference peptide) and the predicted affinity (nM) is shown.

A*0101			
	Peptide	Stability (%)	Affinity (Kd)
High	LTDEMIAQY	102	3
	WTAGAAAYY	88	12
	FNDGVYFAS	49	20788
	NIDGYFKIY	48	1968
Low	CVADYSVLY	25	194
	TSNQVAVLY	15	105
	NSFTRGVYY	12	328
	CNDPFLGVY	6	289

A*0201			
	Peptide	Stability (%)	Affinity (Kd)
High	YLQPRTFLL	106	5
	SIIAYTMSL	105	14
	LLFNKVTLA	104	25
	HLMSFPQSA	102	26
	FLHVTYVPA	102	65
	RLQSLQTYV	98	17
	FQFCNDPFL	69	9
	MIAQYTSAL	67	69
	RLDKVEAEV	66	39
	VLNDILSRL	61	34
Low	KQIYKTPPI	11	86
	FTISVTTEI	29	25
	FIAGLIAIV	10	10
	VTWFHAIHV	25	202
	VLYENQKLI	20	194
	GMSRIGMEV	8	51
	NLNESLIDL	17	177
	VVFLHVTYV	17	37
	WLMWLIINL	6	7
	GLMWLSYFI	5	4

A*0301			
	Peptide	Stability (%)	Affinity (Kd)
High	GVYFASTEK	103	24
	KCYGVSPTK	97	153
	TLKSFTVEK	91	59
	GVYYHKNNK	89	26
	KRFDNPVLP	83	34309
	MTSCCSCLK	83	87
	GIYQTSNFR	80	144
	KSTNLVKNK	73	496
	RLFRKSNLK	71	6
	ASANLAATK	65	154
Low	QIYKTPPIK	38	35
	SVYAWNRKR	7	303
	QIAPGQTGK	6	154
	TLADAGFIK	5	199
	KVFRSSVLH	3	90
	SSTASALGK	2	194
	EILPVSMTK	2	315
	VLKGVKLHY	1	457
	HVSGTNGTK	0	288
	NQFNSAIGK	0	378

Supplementary Table 2: HLA-type for vaccinated donors

Donor	HLA-A		HLA-B		HLA-C		HLA-DRB1	
Vx01	03:01:01G	29:02:01G	39:06:02G	44:03:01G	07:02:01G	16:01:01G	01:01:01G	08:01:01G
Vx02	02:01:01G	32:01:01G	27:02:01G	27:05:02G	02:02:02G	05:01:01G	03:01:01G	11:01:01G
Vx03	01:01:01G	03:01:01G	08:01:01G	15:01:01G	03:04:01G	07:01:01G	03:01:01G	04:01:01G
Vx04	02:01:01G	-	07:02:01G	15:01:01G	07:02:01G	15:05:01G	13:01:01G	15:01:01G
Vx05	01:01	02:01	07:02	18:01	07:02	07:01	11:04:01	15:01:01
Vx06	01:01:01G	03:01:01G	08:01:01G	35:01:01G	04:01:01G	07:01:01G	01:01:01G	03:01:01G

Supplementary Table 3: HLA-type for convalescent donors

Donor	HLA-A		HLA-B		HLA-C		HLA-DRB1	
CV01	01:01:01G	02:01:01G	15:17:01G	27:05:02G	02:02:02G	07:01:01G	01:01:01G	11:01:01G
CV02	01:01:01G	25:01:01G	07:02:01G	08:01:01G	07:01:01G	07:02:01G	03:01:01G	15:01:01G
CV03	01:01:01G	02:01:01G	08:01:01G	15:18:01G	07:01:01G	07:04:01G	01:01:01G	03:01:01G
CV04	03:01:01G	31:01:02G	39:01:01G	55:01:01G	03:03:01G	12:03:01G	11:01:01G	14:54:01G
CV05	01:01:01G	02:01:01G	07:02:01G	52:01:01G	07:02:01G	12:02:01G	13:02:01G	15:02:01G
CV06	02:01:01G	32:01:01G	15:01:01G	18:01:01G	01:02:01G	05:01:01G	03:01:01G	08:01:01G
CV07	01:01:01G	02:01:01G	08:01:01G	15:01:01G	03:03:01G	07:01:01G	01:01:01G	13:01:01G
CV08	29:01:01G	68:01:02G	07:05:01G	18:01:01G	05:01:01G	15:05:01G	11:01:01G	13:01:01G
CV09	01:01:01G	24:02:01G	08:01:01G	27:05:02G	02:02:02G	07:01:01G	04:01:01G	15:01:01G
CV10	02:06:01G	03:01:01G	13:02:01G	37:01:01G	06:02:01G	-	07:01:01G	10:01:01G
CV11	02:01:01G	24:02:01G	27:05:02G	44:02:01G	01:02:01G	05:01:01G	08:01:01G	11:01:01G
CV12	03:01:01G	25:01:01G	18:01:01G	40:01:01G	03:04:01G	12:03:01G	04:01:01G	13:01:01G
